# Polo-like kinase 1 regulates the stability of the mitotic centromere-associated kinesin in mitosis

**DOI:** 10.18632/oncotarget.1861

**Published:** 2014-03-24

**Authors:** Mourad Sanhaji, Andreas Ritter, Hannah R. Belsham, Claire T. Friel, Susanne Roth, Frank Louwen, Juping Yuan

**Affiliations:** ^1^ Department of Gynecology and Obstetrics, School of Medicine, J. W. Goethe-University, Theodor-Stern-Kai 7, D-60590 Frankfurt, Germany; ^2^ School of Life Sciences, University of Nottingham, Medical School, Queen's Medical Centre, Nottingham, NG7 2UH, United Kingdom

**Keywords:** MCAK, Plk1, protein stability, chromosome alignment, spindle assembly

## Abstract

Proper bi-orientation of chromosomes is critical for the accurate segregation of chromosomes in mitosis. A key regulator of this process is MCAK, the mitotic centromere-associated kinesin. During mitosis the activity and localization of MCAK are regulated by mitotic key kinases including Plk1 and Aurora B. We show here that S621 in the MCAK's C-terminal domain is the major phosphorylation site for Plk1. This phosphorylation regulates MCAK's stability and facilitates its recognition by the ubiquitin/proteasome dependent APC/C^Cdc20^ pathway leading to its D-box dependent degradation in mitosis. While phosphorylation of S621 does not directly affect its microtubule depolymerising activity, loss of Plk1 phosphorylation on S621 indirectly enhances its depolymerization activity *in vivo* by stabilizing MCAK, leading to an increased level of protein. Interfering with phosphorylation at S621 causes spindle formation defects and chromosome misalignments. Therefore, this study suggests a new mechanism by which Plk1 regulates MCAK: by regulating its degradation and hence controlling its turnover in mitosis.

## INTRODUCTION

The mitotic spindle is the main cellular tool required for ensuring chromosome segregation and it consists of an array of highly dynamic microtubules (MTs) [1]. Microtubule-associated motor proteins have important functions in spindle assembly. They organize MTs arising from both spindle poles in a fusiform-structure as they promote embedding and clustering MT minus ends in the centrosome and allow MT plus-ends to have dynamic behavior, elongation/shortening, which promotes the capture of kinetochores during the early phase of mitosis [2]. The kinesin-13 family is critically involved in the process of spindle assembly. Unlike other kinesin, this family makes use of the energy produced by ATP-hydrolysis to destabilize MTs from both ends, rather than to move along the MT-surface [3,4]. It encompasses three members, Kif2a [5], Kif2b [6] and MCAK/Kif2c [7] sharing a highly conserved motor domain at the middle of their protein sequence, which is essential for regulating MT dynamics during mitosis and interphase [7,8]. MCAK is the founding and best-characterized member of the kinesin-13 family. During mitosis it is present at different localizations including spindle poles, chromosome arms, centromeres and kinetochores. MCAK is the only member of this family that can be recruited to growing MT plus-ends [9-11]. The activity as well as the subcellular localization of MCAK are tightly regulated by phosphorylation events that are executed by a number of important kinases, like Aurora B, Aurora A, cyclin-dependent kinase 1 (Cdk1) and Polo-like kinase 1 (Plk1) in mitosis [12-17]. Consistent with this, deregulated MCAK is described to cause spindle assembly defects, misaligned chromosomes and aberrant anaphase in mammalian cells [11,12,15,18]. Moreover, several studies set accent on the deregulated status of MCAK in cancer cells and reported an overexpression of this kinesin in several cancer types, notably, in breast, gastric and colorectal cancers [19-21]. Furthermore, deregulated MCAK has been closely linked to cancer invasiveness, metastasis and poor prognosis in these cancer types [19-21].

Plks are a family of serine/threonine kinases involved in the regulation of several cell cycle phases and proliferation [22-25]. Plk1 is the most thoroughly investigated member of this family. Plk1 fulfills various functions that are indispensable for a smooth mitotic progression by interacting with many molecules, such as sororin, which controls loss of chromosome arm cohesion at prometaphase [26,27]. Accordingly, it is found to be present in several subcellular localizations, including centrosomes [28], kinetochores [29] and central spindle where it participates in cytokinesis [30,31]. Plk1 interacts with members of the kinesin-13 family. Together with Aurora A, Plk1 regulates the activity and subcellular localization of Kif2a during mitosis [32]. Additionally, Plk1 associates and phosphorylates Kif2b on S204 and T125 at kinetochores modulating its localization and function [18]. It has been reported that Plk1 promotes the catalytic activity of MCAK by phosphorylating the five residues at its C-terminus, thus regulating MT-dynamics and improving the correction of kinetochore-MT error-attachments [12]. In this study, we have dissected individual phosphorylation of MCAK by Plk1 and characterized its function in more depth. We demonstrate that S621 is the major phosphorylation site of Plk1 on MCAK, which is responsible for regulating MCAK's degradation by promoting the association of MCAK with APC/C^Cdc20^. Interfering with this phosphorylation causes spindle assembly errors and chromosome misalignments. Our data provide a novel mechanism by which Plk1 regulates MCAK in mitosis.

## RESULTS

### MCAK associates with Plk1 in mitosis

**Figure 1:**
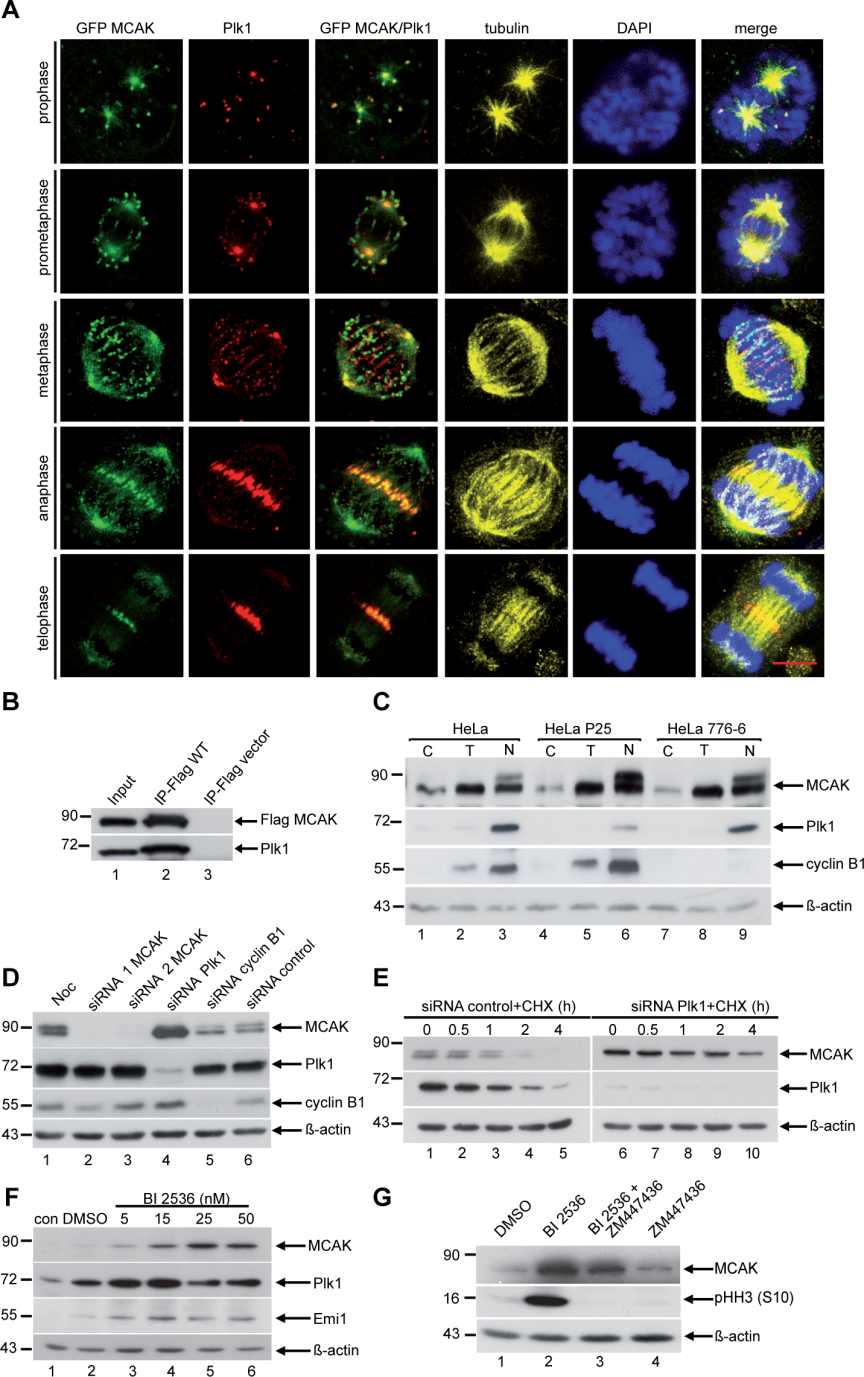
Plk1 interacts with MCAK and downregulation of Plk1 increases the protein level of MCAK in mitosis (A) Co-localization of Plk1 and MCAK by immunofluorescence staining. HeLa cells were transfected with EGFP MCAK construct and synchronized to mitosis. Cells were stained for Plk1, tubulin and DNA. Scale bar: 7.5 µm. (B) Immunoprecipitation was performed using Flag antibody with mitotic extracts from HeLa cells transfected with Flag MCAK. The precipitates were subjected to Western blot analysis with antibodies as indicated. Cellular lysates from Flag-vector transfected cells served as negative control. (C) HeLa, HeLa P25 (Plk1 stably depleted) and HeLa 776-6 (cyclin B1 stably depleted) cells were synchronized to G1/S by the thymidine block (T) or prometaphase by nocodazole (N) or non-treated (C). Cellular lysates were analyzed by Western blot using antibodies as indicated. (D) HeLa cells transfected with control siRNA or siRNA targeting MCAK (siRNA 1 and 2), Plk1 and cyclin B1 were synchronized to prometaphase and cellular lysates were analyzed by Western blot using the antibodies as indicated. (E) HeLa cells were transfected with control siRNA or siRNA against Plk1 and synchronized to prometaphase with nocodazole. The mitotic fraction was collected by shake off and released for the indicated time points in fresh medium containing cycloheximide (CHX, 25 µM). Cellular lysates were prepared for Western blot analyses using the indicated antibodies. (F) HeLa cells were treated with increasing concentrations (5 nM to 50 nM) of the Plk1 inhibitor BI 2536 for 14 h. Cellular lysates were prepared for Western blot analysis with antibodies as indicated. (G) HeLa cells were treated with DMSO, BI 2536 (25 nM) or the Aurora B inhibitor ZM447436 (2 µM) for 14 h. For the combinatory treatment, HeLa cells were first incubated with BI 2536 (25 nM) for 14 h and ZM 447436 (2 µM) was added for the last 2 h of the incubation. Cellular lysates were generated and probed with the indicated antibodies. β-actin served as loading control.

In a previous study of the function of Plk1 in mitosis [33], we observed a link between the Plk1 activity and the protein level of MCAK. To determine the temporal and spatial relationship between Plk1 and MCAK during mitosis, HeLa cells were transfected with EGFP MCAK and stained for endogenous Plk1. Both MCAK and Plk1 were localized to centrosomes and the centromeres/kinetochores in early mitosis (Figure 1A, 1^st^ to 3^rd^ row). In late mitosis, Plk1 and MCAK were recruited to the spindle midzone (Figure. 1A, 4^th^ and 5^th^ row). Moreover, Plk1 and Flag-tagged MCAK co-precipitated from mitotic extracts (Figure. 1B). In addition, MCAK and Plk1 showed a similar expression pattern with a peak at 9-10 h post release from a thymidine block (Figure S1A, 1^st^ and 2^nd^ row). At these time points (9-10 h) MCAK is displayed as double band indicating the existence of a phosphorylated and a non-phosphorylated pool during mitosis. These results suggest a physical interaction of Plk1 and MCAK during mitosis, in accordance with previous reports [12,34].

### Plk1 regulates degradation of MCAK in mitosis

To explore the involvement of Plk1 in the regulation of MCAK protein level, we used HeLa cells depleted of either Plk1 (HeLa P25) [33] or cyclin B1 (HeLa 776-6) [35], compared to HeLa control cells. Cells were synchronized to either the G1/S boundary or to prometaphase and the protein levels were analyzed at different time points during the cell cycle. Compared to control cells, MCAK was obviously stabilized in prometaphase in Plk1 depleted cells (Figure 1C, 1^st^ row, lane 3 compared to lane 6), whereas the level of MCAK increased only slightly in cyclin B1 depleted cells (Figure1C, 1^st^ row, lane 9). To exclude the possibility that increased MCAK level resulted from an unspecific effect of the stable knockdown of Plk1, HeLa cells were transiently transfected with siRNA targeting Plk1, synchronized to mitosis and the protein level of MCAK was monitored. Upon depletion of Plk1, MCAK accumulated as compared to cells transfected with siRNA against cyclin B1 or control siRNA (Figure 1D, 1^st^ row, lanes 4, 5 and 6).

To determine if increased levels of MCAK resulted from inhibition of degradation, HeLa cells were synchronized to prometaphase and released in fresh medium in the presence of cycloheximide (CHX), a protein synthesis inhibitor. Cells were harvested at the indicated time points and protein levels were determined. In cells transfected with control siRNA, degradation of MCAK began about 1 h post release (Figure 1E, 1^st^ row, lanes 1-5). However, in cells depleted of Plk1 MCAK levels remained constant for about 2 h and a considerable level of MCAK was still detected at 4 h (Figure 1E, 1^st^ row, lanes 6-10), a time point at which MCAK was not detectable in control cells (Figure 1E, 1^st^ row, lane 5).

To study if the kinase activity of Plk1 is involved in MCAK degradation, we added the compound BI 2536, a selective and potent inhibitor of Plk1 [36], to our assay. The kinase activity of Plk1 in HeLa cells was efficiently inhibited, as demonstrated by an accumulation of the protein Emi1 (Figure 1F, 3^rd^ row, lanes 3-6), which is degraded in early mitosis in response to Plk1 phosphorylation [37]. Inhibition of Plk1 kinase activity resulted in stabilization of MCAK in mitotic HeLa cells in a dose dependent manner (Figure 1F, 1^st^ row, lanes 3-6), suggesting that turnover of MCAK is related to the activity of Plk1 in mitosis.

It has been reported that depletion or inhibition of Plk1 leads to a pronounced mitotic arrest as a result of activation of the spindle assembly checkpoint (SAC) [23,36,38,39]. Therefore, we asked whether the observed MCAK accumulation in response to Plk1 inhibition is a direct effect or a consequence of mitotic arrest. To test this, cells arrested by the presence of the potent Plk1 inhibitor BI 2536 were treated with ZM447436, an Aurora B inhibitor, which inactivates the SAC [40]. Cells treated with BI 2536 alone arrested in mitosis shown by an increased signal of phospho-histone H3 (pHH3 (S10)) (Figure 1G, 2^nd^ row, lane 2). This was accompanied by a substantial accumulation of MCAK compared to DMSO or ZM447436 only treated cells (Figure 1G, 1^st^ row, lanes 1, 2 and 4). Cells incubated with the combination of BI 2536 and ZM447436 exited mitosis, evidenced by an absence of the pHH3 (S10) signal (Figure 1G, 2^nd^ row, lane 3), comparable to ZM447436 only treated cells (Figure 1G, 2^nd^ row, lane 4). Nevertheless, in these cells MCAK still accumulated relative to ZM447436 only treated cells (Figure 1G, 1^st^ row, lanes 3 and 4). This observation indicates that the accumulation of MCAK observed in response to Plk1 inhibition is not a consequence of mitotic arrest, but can be directly ascribed to the inhibition of Plk1. Collectively, these results indicate that Plk1's kinase activity influences the turnover of MCAK in mitosis.

### The major Plk1 phosphorylation site is S621 in MCAK's C-terminal domain

**Figure 2:**
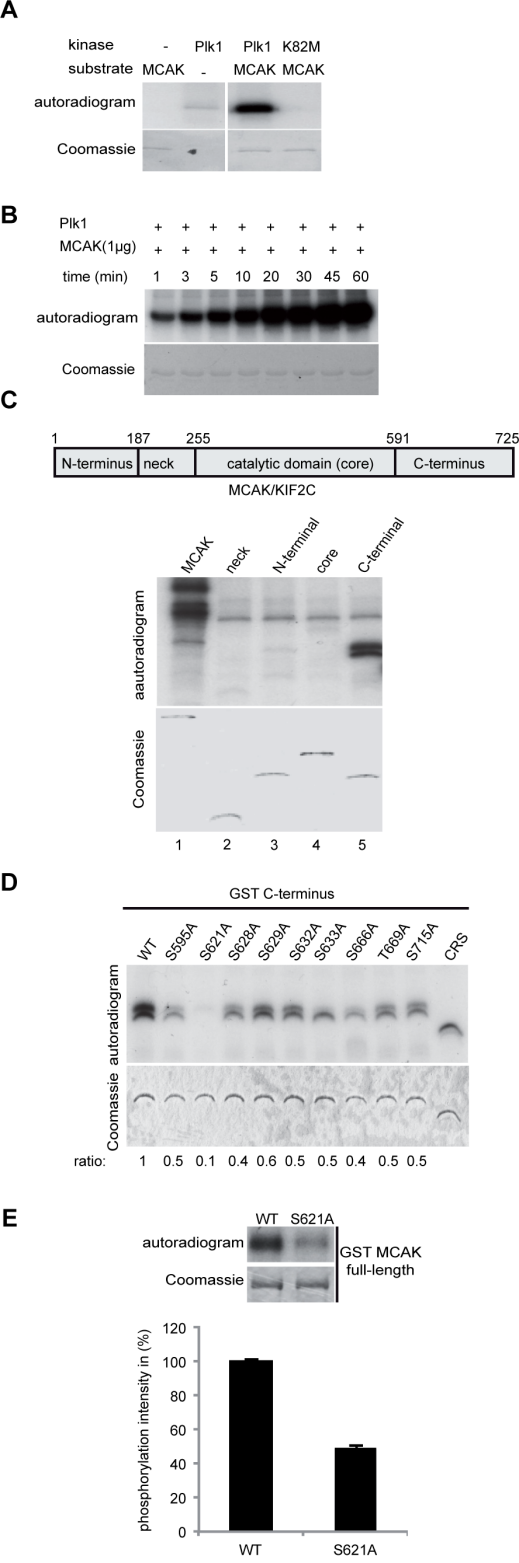
Plk1 phosphorylates MCAK on its C-terminus in vitro (A) Purified his_6_-tagged MCAK was subjected to kinase assays *in vitro* with active Plk1 and kinase dead form Plk1 K82M. (B) Time kinetics of MCAK phosphorylation by Plk1. (C) Upper panel: schematic illustration of various domains of MCAK. Lower panel (autoradiogram): kinase assays *in vitro* of GST-tagged MCAK domains with Plk1. The same gel was stained with Coomassie and used as loading control (Coomassie). (D) *In vitro* kinase assay of recombinant GST-MCAK C-terminus and its mutants. Phosphorylated MCAK C-termini by Plk1 were visualized by autoradiography (upper panel). The same gel was stained with Coomassie as loading control (lower panel). The phosphorylation ratio is indicated at the bottom of the Coomassie gel. The values were calculated in relation to the C-terminus wild type (WT=1). (E) *In vitro* kinase assay of recombinant full-length GST MCAK and GST MCAK S621A using Plk1 kinase. The quantification of Plk1 phosphorylation was performed using the Image J software. The results are presented as mean ± SD (*n*=3).

To verify that Plk1 phosphorylates MCAK, we performed an *in vitro* kinase assay with purified His-tagged full-length MCAK as the substrate. In this assay Plk1, but not its kinase-deficient variant Plk1 K82M, readily phosphorylated the His-tagged MCAK (Figure 2A), confirming that MCAK is a substrate of Plk1 *in vitro*. Furthermore, the degree of phosphorylation increased in a time-dependent manner (Figure 2B). To map the location of the Plk1 phosphorylation site(s) in MCAK, we performed the *in vitro* kinase assay on various GST-tagged MCAK constructs (Figure 2C). Only the C-terminal domain of MCAK was clearly phosphorylated by Plk1 (Figure 2C, lane 5), in line with previous reports [12,34]. We then individually mutated each serine/threonine in the C-terminal domain only construct to alanine and performed the kinase assay. Mutation of S621 almost entirely abolished incorporation of ^32^P and to a much greater extent than mutation of any other serine/threonine (Figure 2D). Furthermore, the mutation S621A in full-length GST-MCAK decreased the phosphorylation intensity to 50% relative to the wild-type (Figure 2E). These data confirm the existence of multiple Plk1 phosphorylation sites on MCAK's C-terminus [12] but highlight S621 as the major Plk1 phosphorylation site.

### The APC/CCdc20 pathway regulates MCAK degradation

**Figure 3:**
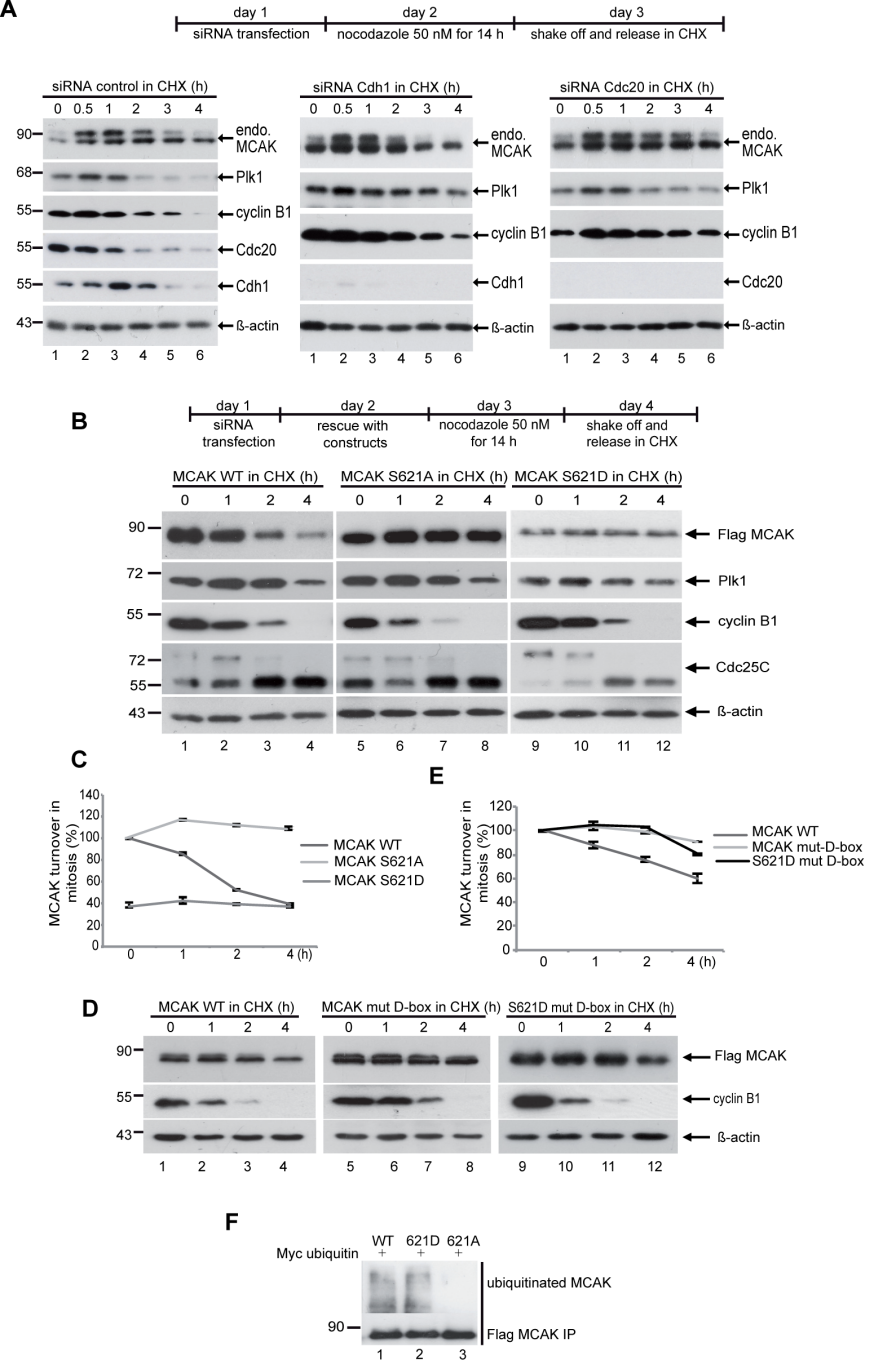
The degradation of MCAK is proteasome dependent via the APC/C Cdc20 and Plk1 phosphorylation of S621 is required for mitotic degradation of MCAK (A) HeLa cells were transfected with control siRNA, siRNA against Cdc20 or Cdh1 and synchronized to prometaphase with nocodazole (50 ng/ml). Mitotic cells were collected by shake off and incubated in fresh medium containing CHX (25 µM) for the indicated times. Cellular lysates were analyzed by Western blot using the indicated antibodies. (B) HeLa cells were transfected with Flag MCAK WT, the non-phosphorylatable variant MCAK S621A, the phosphomimetic variant MCAK S621D upon depletion of endogenous MCAK using siRNA targeting its 3′-untranslated region. Cells were synchronized to prometaphase, released into fresh medium containing CHX and harvested at the indicated time points. Cellular lysates were analyzed by Western blot using antibodies as denoted. β-actin served as loading control. (C) Quantification was performed based on three independent experiments using the Image J software. The results are presented as mean ± SD (*n*=3). (D) Mutation of the D-box prevents the degradation of MCAK during mitosis. D-box mutant MCAK (MCAK WT mut-D-box) and the phosphomimetic variant bearing a modified D-box (MCAK S621D mut-D-box) have been added back to HeLa cells after knockdown of endogenous MCAK using siRNA targeting its 3'-untranslated region. Cells were synchronized to prometaphase, released into fresh medium containing CHX and harvested at the indicated time points. Cellular lysates were analyzed with Western blot using the annotated antibodies. β-actin served as loading control. (E) The quantification was performed using the Image J software. The results are presented as mean ± SD (*n*=2). (F) Preventing the phosphorylation of S621 by Plk1 suppresses the ubiquitination of MCAK during late mitosis. HeLa cells were transfected with Flag MCAK WT and its mutants and synchronized to prometaphase. The mitotic fraction was collected by shake off and released in fresh medium containing the proteasome inhibitor MG132 (10 µM) for 2 h. Cell lysates were subjected to immunoprecipitation with Flag antibody. The precipitates were separated by Western blot and stained with ubiquitin antibody.

Having shown that MCAK is phosphorylated by Plk1 and that this phosphorylation influences degradation of MCAK during mitosis, we next asked whether degradation of MCAK occurs via one or both of the ubiquitin/proteasome dependent pathways APC/C^Cdc20^ and APC/C^Cdh1^. These pathways are responsible for degradation of many proteins during mitosis, such as securin and cyclin B1 [41-43]. To address this question, HeLa cells treated with siRNA targeting Cdc20 or Cdh1 were synchronized to prometaphase, released in the presence of a protein synthesis inhibitor CHX and protein levels of endogenous MCAK were monitored over time (Figure 3A). In cells treated with either control siRNA or siRNA against Cdh1, the levels of phosphorylated MCAK (slow migrating band) began to decrease at 2 h post mitotic release (Figure 3A, left and middle panels, 1^st^ rows) and shortly after the degradation of cyclin B1 (Figure 3A, left and middle panels, 3^rd^ rows). In these cells, almost no phosphorylated MCAK was detected 4 h post release. By contrast, in cells depleted of Cdc20 the level of phosphorylated MCAK remained constant up to 3 h post release and a significant level was still detectable at 4 h post release (Figure 3A, right panel, 1^st^ row). This shows that knockdown of Cdc20 increases the stability of phosphorylated MCAK and strongly suggests that APC/C^Cdc20^ is involved in the degradation of MCAK during mitosis.

### Phosphorylation at S621 is required for mitotic degradation of MCAK

As Cdc20, required for the turnover of mitotic cyclins [44], is essential for promoting the activation of the anaphase-promoting-complex [45], depletion of Cdc20 leads to a delay in the metaphase-anaphase transition. To exclude the possibility that the stabilization of endogenous MCAK (Figure 3A) is due to the delayed transition as well as to test if the phosphorylation of S621 is linked to degradation of MCAK in mitotic cells, we used HeLa cells depleted of endogenous MCAK and rescued with Flag-tagged versions of MCAK: either the wild-type (WT), the non-phosphorylatable variant S621A or the phosphomimetic form S621D. Cells were synchronized to prometaphase and shake-off cells were reseeded in CHX containing medium to inhibit protein synthesis (Figure 3B). Levels of protein were measured up to 4 h post release (Figure 3B, upper panel). MCAK WT began to decrease 2 h after release and was reduced to ~50% by 4 h post release (Figure 3B, 1^st^ row, lanes 1-4, and C), concomitant with cyclin B1 degradation (Figure 3B, 3^rd^ row, lanes 1-4). By contrast, the level of non-phosphorylatable MCAK S621A remained stable throughout mitosis and even after cells entered the subsequent G1 phase (Figure 3B, 1^st^ row, lanes 5-8, and C). Levels of the phosphomimetic MCAK S621D were low upon release and remained consistently low at all points post release (Figure 3B, 1^st^ row, lanes 9-12, and C). This remaining pool of non-degradable MCAK, even in the case of the phosphomimetic S621D, perhaps suggests a threshold below which MCAK is not degraded or a subcellular pool of MCAK not regulated by this phosphorylation. Together these results suggest that phosphorylation of S621 by Plk1 promotes degradation of MCAK at the transition from metaphase to anaphase likely via generation of a degradation competent conformation as a result of phosphorylation.

### A “D-box” motif found in MCAK's neck domain is required for mitotic degradation

Our data suggests that phosphorylation at S621 targets MCAK for degradation via the APC/C^Cdc20^ proteasome. Mitotic proteins degraded by the APC/C^Cdc20^ pathway generally carry a degradation box (D-box) motif [46,47]. The D-box consensus sequence is RxxLxxxxN/E/D, where x is any amino acid. However, most substrates contain only the highly conserved core RxxL motif, which is sufficient for recognition [46,47]. Sequence analysis of MCAK reveals the presence of a D-box core motif RATL (aa 240-243) in the neck domain. We asked if this D-box is required for turnover of MCAK via APC/C^Cdc20^. HeLa cells depleted of endogenous MCAK were rescued with Flag-tagged MCAK WT or a variant with the D-box mutations R240A/L243A. Cells were synchronized to prometaphase, released in CHX containing medium and MCAK levels measured at the indicated time points. As observed previously (Figure 3B), MCAK WT levels began to decrease at 2 h post release (Figure 3D, 1^st^ row, lanes 1-4, and E). By contrast levels of MCAK R240A/L243A remained constant up to 4 h post release (Figure 3D, 1^st^ row, lanes 5-8, and E). Introducing these D-box mutations in the background of the phosphomimetic variant of MCAK, S621D, also protects this variant from degradation (Figure 3D, 1^st^ row, lanes 9-12, and E). These data suggest that the D-box motif is necessary for degradation of MCAK during mitosis. Abolishing this motif by mutagenesis not only stabilized WT MCAK but also the phosphomimetic variant S621D.

HeLa cells overexpressing Flag MCAK WT, MCAK S621A or MCAK S621D and myc-tagged ubiquitin were synchronized to prometaphase and released in medium containing the proteasome inhibitor MG132. These cellular lysates were then subjected to immunoprecipitation with a Flag antibody. Flag MCAK WT and Flag MCAK S621D showed significant levels of ubiquitination (Figure 3F, upper panel, lanes 1 and 2). However, Flag MCAK S621A showed no significant ubiquitination suggesting that it cannot be targeted to the APC/C^Cdc20^ degradation pathway (Figure 3F, upper panel, lane 3). Notably, in the presence of MG132, the level of MCAK S621D was comparable with that of WT and S621A (Figure 3F, lower panel), indicating a proteasome dependence of MCAK's degradation. Taken together, these data show that phosphorylation of MCAK by Plk1 at residue S621 facilitates its degradation in mitosis by the ubiquitin/proteasome dependent APC/C^Cdc20^ pathway.

### Mutations S621A and S621D do not affect the catalytic activity of MCAK

**Figure 4:**
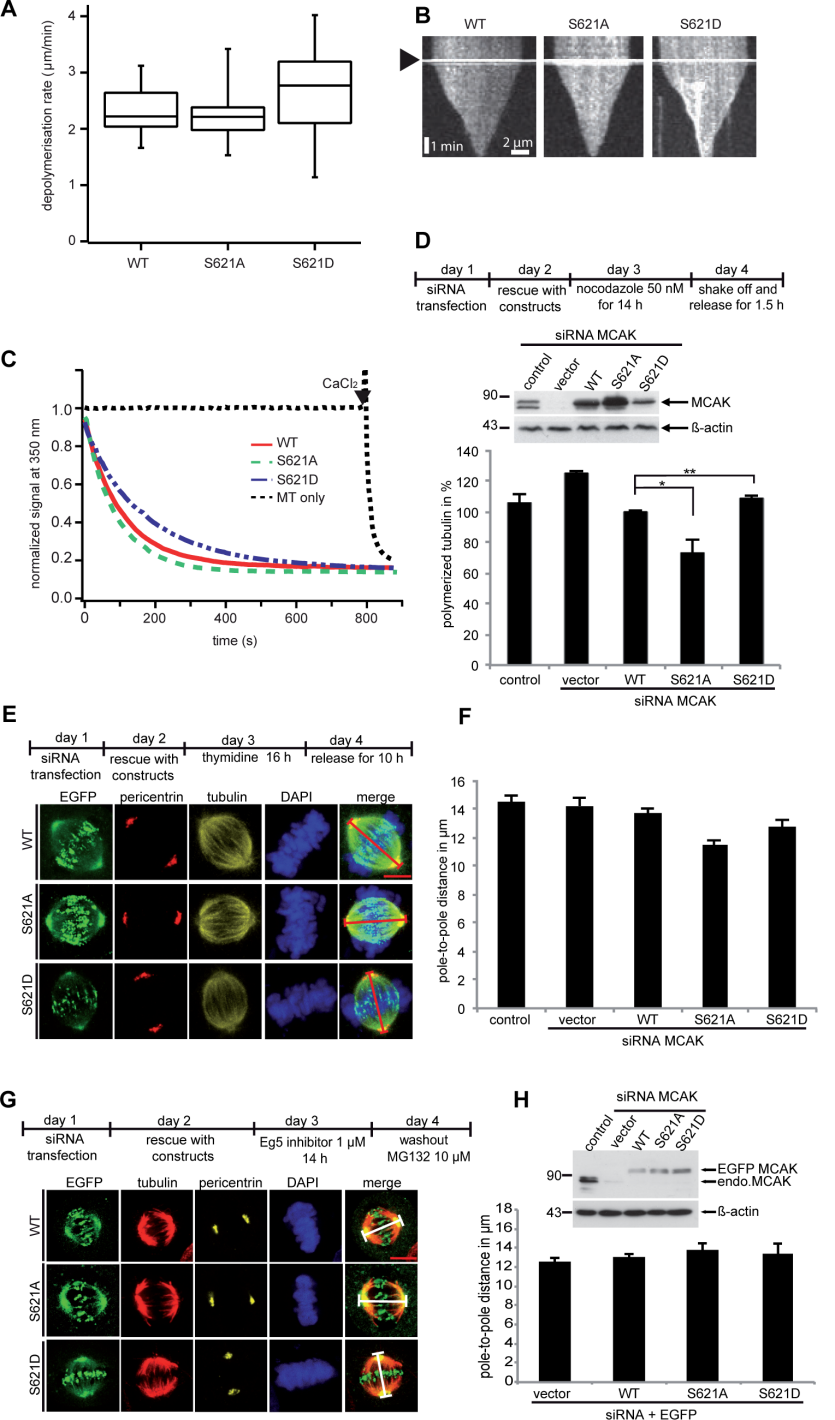
Mutation at S621 does not significantly affect the microtubule depolymerizing activity of MCAK (A) *In vitro* depolymerization assays. Box plots depicting the distribution of depolymerization rates of individual microtubules monitored using fluorescence microscopy for MCAK WT (n=22), MCAK S621A (n=22) or MCAK S621D (n=23). (B) Kymographs of representative microtubules depolymerized by MCAK WT, S621A or S621D. Arrowhead indicates addition of MCAK. (C) *In vitro* microtubule depolymerization monitored by light scattering at 350 nm. (D) Measurement of polymerized tubulin content in HeLa cells. Upper panel: working schedule. HeLa cells were transfected with Flag-tagged MCAK WT, its mutants or the empty vector in an endogenous MCAK depleted background. Transfected cells were then synchronized to prometaphase and released for 1.5 h. Middle panel: Western blot analyses as transfection controls. Lower panel: cellular polymerized tubulin contents were analyzed by flow cytometry after cells were extracted, fixed and stained for tubulin. The amount of polymerized tubulin from Flag MCAK WT-transfected HeLa cells was assigned as 100%. The results are presented as mean ± SD (*n*=3). **P < 0.01, *P < 0.05. (E) Measurement of the pole-to-pole distance. Upper panel: working schedule. HeLa cells were transfected with EGFP-tagged MCAK WT, MCAK S621A, MCAK S621D or EGFP vector after depleting endogenous MCAK with siRNA. Cells were synchronized to G1/S and released for 10 h. Lower panel: examples of metaphase cells rescued with EGFP MCAK and its mutant EGFP MCAK S621A and EGFP MCAK S621D. Scale bar: 7.5 µm. (F) The pole-to-pole distance was measured using the LAS AF software (n=24 metaphase cells for each condition). The results are presented as mean ± SD and statistically analyzed. ***p < 0.001. (G) Upper panel: working schedule. HeLa cells were depleted of endogenous MCAK and followed by rescue with EGFP MCAK and its mutants 24 h prior to treatment with Eg5 inhibitor III (0.8 µM) for 14 h. The inhibitor was washed out and cells were released for 1 h in fresh medium containing MG132 (10 µM) to retain cells at metaphase and stained for tubulin, pericentrin and DNA. Lower panel: examples of metaphase cells rescued with EGFP MCAK and its mutant EGFP MCAK S621A and EGFP MCAK S621D. Scale bar: 7.5 µm. (H) Upper panel: Western blot analysis of the expression levels of EGFP MCAK and its mutants in HeLa cells upon rescue. Lower panel: quantification of the polo-to-pole distance, measured using the LAS AF software. The results are presented as mean ± SD (n=25 metaphase cells for each condition).

To determine the effect of phosphorylation at S621 on the activity of MCAK, his_6_-tagged MCAK WT, S621A and S621D were expressed and purified. The ability of MCAK and variants to depolymerize microtubules was investigated using an *in vitro* depolymerization assay [48]. Neither the mutation S621A nor S621D significantly impacted the depolymerization activity of MCAK *in vitro*. The MT depolymerization rates were 2.3±0.4, 2.3±0.5 and 2.6±0.8 µm/min for WT, S621A and S621D, respectively (Figure 4A and B). This result was corroborated using light scattering to follow MT depolymerization. Both MCAK S621A and MCAK S621D readily depolymerized MTs (Figure 4C). These data suggest that phosphorylation at S621 does not directly affect the catalytic activity of MCAK. Furthermore, the basal rate of ATP turnover is not significantly different for MCAK WT, MCAK S621A and MCAK S621D: 2.1±0.7×10^−3^, 3.0±0.6×10^−3^ and 3.2±0.8×10^−3^ s^−1^, respectively (Figure S1B), indicating that these mutations have not disrupted the conformation of the motor domain nor of the nucleotide-binding site.

To investigate the microtubule depolymerization activity of MCAK WT and its mutants *in vivo*, HeLa cells were depleted of endogenous MCAK and replaced 24 h later by Flag-tagged MCAK WT, MCAK S621A or MCAK S621D (Figure 4D, upper panel). Endogenous MCAK was efficiently depleted using siRNA (Figure 4D, middle panel). Varying amounts of the Flag-tagged MCAK and variants were observed (Figure 4D, middle panel) consistent with the differential degradation suggested by our previous data. The unphosphorylatable S621A showed higher levels than WT MCAK; whilst the phosphomimetic S621D displayed a reduced level compared to WT. Cells transfected with the non-degradable variant S621A contained 25% less polymerized tubulin than cells transfected with MCAK WT (Figure 4D, lower panel). By contrast, cells expressing the rapidly degraded form MCAK S621D contained 8% more polymerized tubulin relative to MCAK WT cells (Figure 4D, lower panel). Similar observations were also obtained in HCT116 cells (Figure S2A). Since *in vitro* assays show no differential catalytic activity between these variants and WT-MCAK, the different amounts of polymerized tubulin observed likely result from different protein levels of these variants due to their differential degradation. The pole-to-pole distance in cells rescued with the degradation resistant variant MCAK S621A was shorter than in cells transfected with MCAK WT or MCAK S621D (Figure 4E and F), corroborated in HCT116 cells (Figure S2B and C), consistent with higher protein levels of S621A resulting in increased microtubule depolymerization.

To confirm the hypothesis that different protein levels of MCAK WT, S621A and S621D are responsible for the observed differences in amount of tubulin polymer in cells expressing these constructs, HeLa cells were rescued with EGFP-tagged MCAK and its variants after depletion of endogenous MCAK. Cells were then treated with the Eg5 Inhibitor III for 14 h, which generates monopolar spindles without affecting microtubule dynamic and microtubule polymer level [49]. Cells were released for 25 min in fresh medium containing MG132. Arresting cells in metaphase suppresses degradation of MCAK S621D (Figure 4G, upper panel) allowing us to measure the pole-to-pole distance in cells containing similar levels of MCAK variants (Figure 4H, upper panel). In this assay cells expressing MCAK WT, S621A or S621D had spindles with comparable pole-to-pole distances (Figure 4G and H). This is consistent with *in vitro* data showing no significant difference in catalytic activity between MCAK WT or these variants and indicates that differences in the amount of polymerized tubulin (Figure 4D) as well as in spindle length (Figure 4F) can be ascribed to the different levels of MCAK in these cells.

### Expression of the phosphomimetic S621D results in reduced inter-kinetochore distance

**Figure 5:**
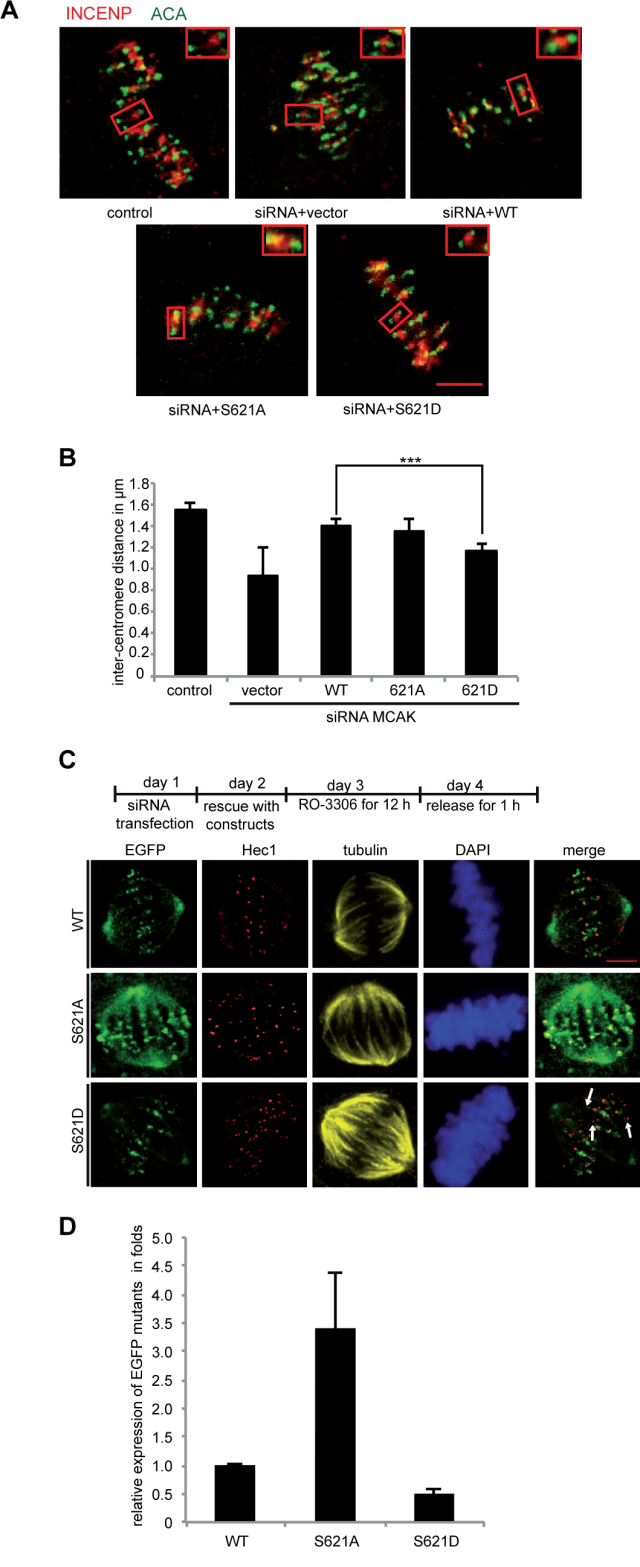
Inter-centromere distance is reduced in mitotic cells expressing MCAK S621D (A) Representatives of the measurement of the inter-centromere distance in HeLa cells. HeLa cells depleted of endogenous MCAK were rescued with Flag MCAK and its mutants and synchronized to mitosis after 12 h release from a double thymidine block. Cells were stained for tubulin, INCENP and ACA. Examples of measured inter-centromere distance in HeLa cells rescued with Flag MCAK, Flag MCAK S621A and Flag MCAK S621D are represented. Scale bar: 7.5 µm. (B) The inter-centromere distance was measured using the LAS AF software (n=55 kinetochore pairs in 15 metaphase cells for each condition). The results are presented as mean ± SD and statistically analyzed. ***p < 0.001. (C) Upper panel: working schedule. HeLa cells were transfected with siRNA targeting only endogenous MCAK followed by the rescue with EGFP MCAK WT and its mutants MCAK S621A and MCAK S621D. Cells were synchronized to the G2 phase using the Cdk1 inhibitor RO-3306 and released in fresh medium for 1 h. Transfected cells were stained for Hec1, tubulin and DNA. Lower panel: representatives of HeLa cells expressing MCAK and its mutants. Scale bar: 12.5 µm. (D) Quantification of the relative expression of MCAK WT and its mutants in synchronized HeLa cells. The data is represented as mean folds ± SD (n= 10 metaphase cells for each condition). ***p < 0.001

It is well established that MCAK is involved in generating tension between sister kinetochores [17,50] and the inter-kinetochore distance is a reliable indicator of this tension. HeLa cells depleted of endogenous MCAK and transfected with Flag MCAK variants were synchronized to the G1/S boundary and released for 12 h to reach late mitosis. Cells were then stained for INCENP and ACA (anti-centromere antibody), the centromere and kinetochore markers (Figure 5A and B). Upon depletion of MCAK, the inter-kinetochore distance was decreased. In cells transfected with MCAK WT or S621A the inter-kinetochore distance was somewhat restored, indicating that MCAK WT and MCAK S621A have similar activity at the centromere/kinetochore region. By contrast, cells transfected with MCAK S621D had a significantly reduced inter-kinetochore distance, relative to MCAK WT expressing cells.

To determine if this reduced inter-kinetochore distance in cells expressing MCAK S621D was due to a low level of this protein as a result of increased degradation, HeLa cells were depleted of endogenous MCAK and rescued with EGFP MCAK and variants. Cells were then synchronized to the G2 phase using the selective Cdk1 inhibitor RO-3306 [51] and released in fresh medium for 1 h (Figure 5C, upper panel). Quantification of the relative expression of EGFP MCAK showed an accumulation of MCAK S621A (Figure 5C and D). These cells contained 3.4 fold more MCAK S621A relative to cells expressing MCAK WT, corroborating the notion that this mutation prevents the degradation of MCAK during mitosis. In contrast, MCAK S621D was found at only 50% of MCAK WT level (Figure 5C and D), substantiating the notion that Plk1 phosphorylation facilitates its degradation. This low level of MCAK S621D in fact appears to result in the absence of MCAK from some centromere/kinetochores, revealed by absence of EGFP signal at sites stained by the centromere marker Hec1 (Figure 5C, 3^rd^ panel, white arrow). Given that both S621A and S621D have been shown to have similar catalytic activity relative to MCAK WT, the reduced inter-kinetochore distance in cells expressing MCAK S621D is likely a consequence of the rapid degradation of this mutant.

### MCAK S621 mutants generate abnormal spindle and chromosome displacement

**Figure 6:**
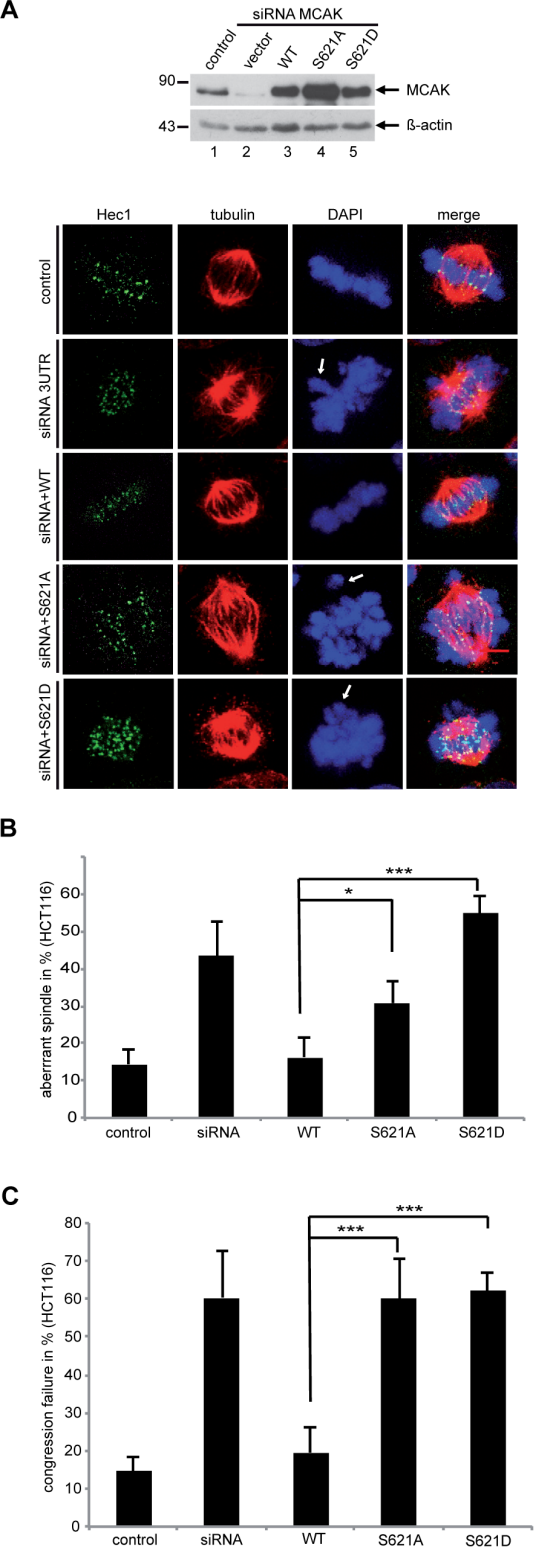
MCAK S621A and MCAK S621D cause defects in spindle formation and chromosome congression Rescue experiment: HCT116 cells were transfected with siRNA targeting only endogenous MCAK followed by the rescue with Flag MCAK wild type and its mutants Flag MCAK S621A and Flag MCAK S621D. Cells were synchronized to G1/S by a double thymidine block and released into fresh medium for 12 h. Transfected cells were stained for tubulin and DNA and morphology of spindle and chromosome in metaphase cells were evaluated. (A) Upper panel: Western blot analysis as controls. Lower panel: Examples of aberrant spindles and chromosome mispositioning in HCT116 cells transfected with siRNA against endogenous MCAK and rescued with Flag MCAK and its mutants. White arrows: misaligned chromosomes. Scale bar: 7.5 µm. (B) The percentage of cells showing defective or aberrant spindles (n=400-600 mitotic cells in each condition). The results are represented as mean ± SD and statistically analyzed. *p < 0.05, ***p < 0.001. (C) The frequency of chromosome mispositioning defined microscopically using DAPI stain (n=400-600 mitotic cells in each condition). The data are displayed as mean ± SD and statistically analyzed. ***p < 0.001.

Finally, to examine the effect of phosphorylation at S621 *in vivo*, we studied the phenotype generated by the expression of MCAK WT, S621A and S621D in mitotic HCT116 cells depleted of the endogenous protein. Cells were stained for tubulin and Hec1, a centromere marker. Spindle morphology and chromosome positioning at metaphase were evaluated by immunofluorescence microscopy. 43% of MCAK depleted cells displayed the typical “hairy” phenotype, forming bipolar spindles but with dense and excessively long and disorganized microtubules (Figure 6A, 2^nd^ panel, tubulin staining, and B), as previously reported [52-54]. Furthermore, 60% of MCAK depleted cells showed defects in chromosome positioning, displaying single or several chromosomes not aligned onto the metaphase plate (Figure 6A, 2^nd^ panel, DAPI staining, white arrow, and C). Expression of WT MCAK reduced the proportion of cells showing spindle aberration back to control levels: 16% for MCAK WT relative to 14% for control cells (Figure 6B). Expression of MCAK WT also reduced the percentage of cells displaying incorrectly aligned chromosomes to 19% in metaphase, comparable with the control cell level of 15% (Figure 6C).

Expression of MCAK S621A reduced spindle formation errors from 43% observed in the knock down to 30% (Figure 6B). However, S621A did not fully restore spindle aberrations to the level displayed by cells expressing MCAK WT. Expression of the phosphomimetic MCAK variant S621D did not restore levels of spindle formation errors. Of cells expressing MCAK S621D, 55% showed aberrant spindles (Figure 6B), which is actually slightly higher than cells depleted of MCAK (Figure 6B). Neither expression of S621A nor S621D could restore congression errors with 60% for S621A and 62% for S621D of metaphase cells showing misaligned chromosomes compared to 19% in cells expressing MCAK WT and 15% in control cells (Figure 6A, white arrows, and C). Thus, neither MCAK S621A nor MCAK S621D could fully restore the phenotype induced by depleting endogenous MCAK. Comparable results were also observed in HeLa cells (Figure S3).

## DISCUSSION

Since unregulated levels of MCAK are associated with cancer invasiveness, metastasis and poor prognosis of patients [55], it is important to delineate the regulatory mechanisms responsible for MCAK's turnover. It has been reported that phosphorylation at S628 and S629 in MCAK is linked to MCAK degradation [34]. However, in our *in vitro* kinase assays, we observed neither S628 nor S629 to be significantly phosphorylated, suggesting that these residues are not the major targets for Plk1.

In this study, we demonstrate that S621 is the major phosphorylation site of MCAK by Plk1 and show that this phosphorylation facilitates MCAK's degradation. A previous study identified five Plk1 phosphorylation sites in MCAK's C-terminal domain: S592, S595, S621, S633 and S715 [12]. Since Aurora B phosphorylates different residues of MCAK at different mitotic stages for different functions [14], it would be meaningful to determine the effect of phosphorylation at individual Plk1 sites. We therefore dissected the Plk1 phosphorylation sites within MCAK. We found that S621 is the major phosphorylation site for Plk1 and so investigated this site in detail. Our data suggest that phosphorylation of MCAK at S621 by Plk1 regulates its degradation throughout mitosis. We observe degradation of MCAK beginning at the metaphase to anaphase transition (Figure 3A), consistent with a previous report [34], and simultaneously with the turnover of cyclin B1. Interfering with the phosphorylation of MCAK at S621 by inhibiting Plk1 leads to stabilization of MCAK in mitosis (Figure 1C-G). Depletion of the APC/C cofactor Cdc20 resulted in enhanced MCAK steady state levels and prevented its degradation in mitotic cells relative to control cells or cells depleted of another APC/C cofactor Cdh1 (Figure 3A). Furthermore, both wild type and the phosphomimetic variant of MCAK, S621D, were protected from degradation by mutation of the D-box motif found in the neck domain of MCAK (Figure 3D). It remains to be clarified if phosphorylation of MCAK by Aurora B also affects the mitotic turnover of MCAK. Nevertheless, these data indicate that phosphorylation at S621 by Plk1 targets MCAK for degradation via the APC/C^Cdc20^ pathway.

**Figure 7:**
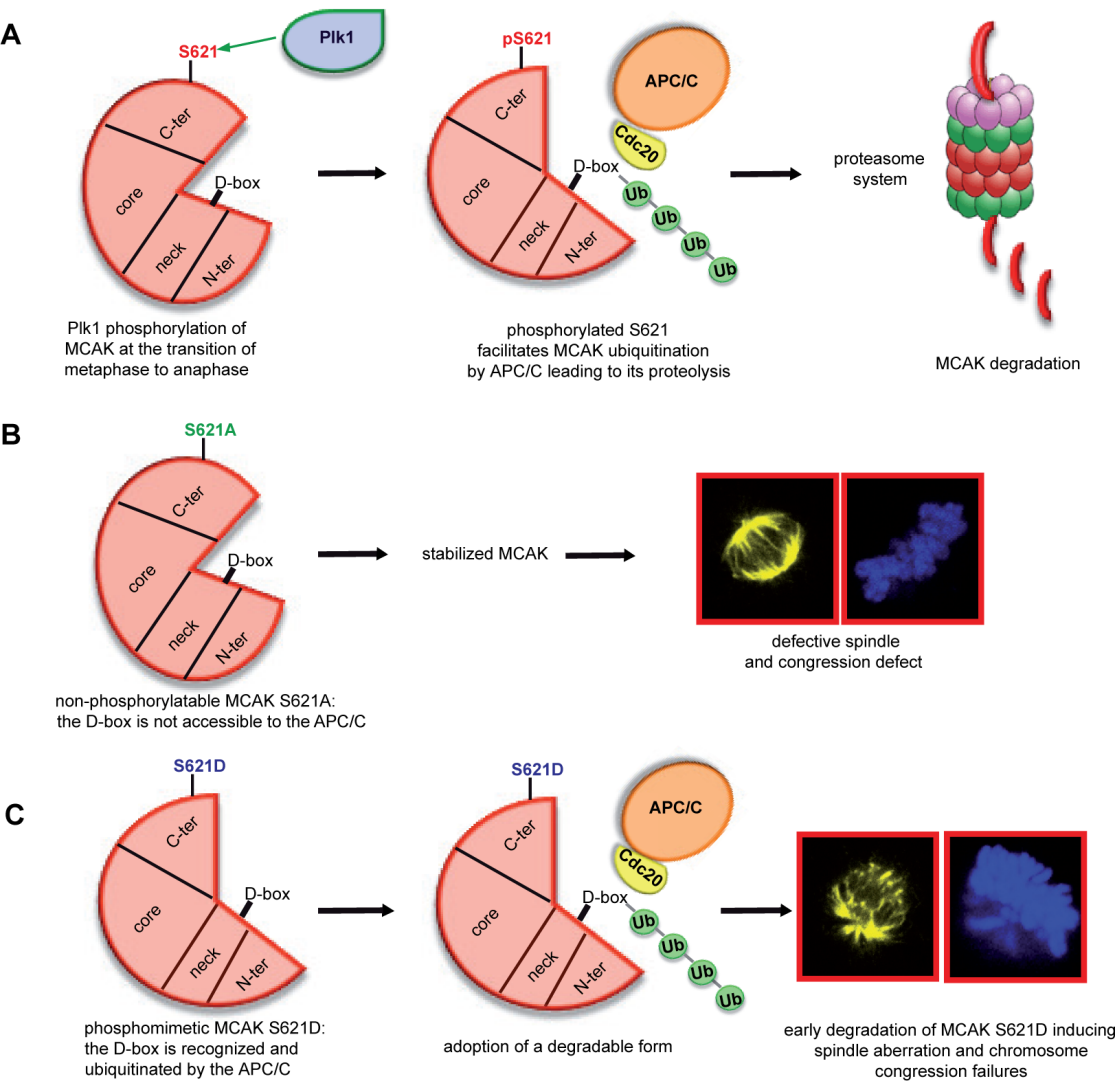
Illustration of S621 phosphorylation by Plk1 facilitating the turnover of MCAK (A) Plk1 phosphorylation of MCAK at S621 makes the D-box accessible to the APC/C^Cdc20^ leading to its ubiquitination and degradation in a proteasome dependent manner. (B) Preventing this phosphorylation by changing serine to alanine stabilizes MCAK during mitosis leading to the assembly of shorter and defective spindles accompanied by chromosome misalignments. (C) The phosphomimetic form MCAK S621D, which displays a degradation competent form, is rapidly degraded causing the formation of aberrant spindle and defects in chromosome positioning.

MCAK plays an important role in the correction of kinetochore-microtuble mal-attachments, thus facilitating the transition from metaphase to anaphase. We speculate that, after accomplishment of its error correction function at metaphase, phosphorylation of MCAK at S621 by Plk1 causes a conformational change resulting in the D-box localized in the neck domain becoming accessible to APC/C^Cdc20^. We suggest that this conformational change is the means by which phosphorylation by Plk1 targets MCAK for degradation. Accordingly, the non-phosphorylatable MCAK mutant S621A accumulates in mitotic cells, whereas the phosphomimetic S621D is only present at low levels suggesting rapid degradation (Figure 3B and C). We also observed that MCAK WT and MCAK S621D can be readily ubiquitinated, whilst MCAK S621A is not significantly ubiquitinated (Figure 3F). Collectively, these data suggest that phosphorylation at S621 by Plk1 causes a conformational change in MCAK, which is required for the APC/C^Cdc20^ to recognize the D-box motif, localized in the neck domain, and target MCAK for degradation (Figure 7A). This conformational change introduced by Plk1 phosphorylation is in line with a previous study unveiling an interplay between the C-terminus and the N-terminal domains of MCAK [56]. Moreover, it has been recently shown that Aurora B phosphorylation at S196 in the MCAK's neck domain generates an ‘open’ conformation regulating the activity of MCAK by disrupting the interaction between the neck and the C-terminal domains [57]. Phosphorylation by Plk1 at S621 in MCAK's C-terminal domain may have a similar effect: disrupting the interaction of the C-terminus with the neck domain and revealing the D-box motif, which targets MCAK for degradation.

Unlike Aurora B or Cdk1 [15,16], phosphorylation of the five Plk1 sites, identified by Zhang et al, activates MCAK's catalytic activity during the early phases of mitosis [12]. Interfering with phosphorylation of these residues resulted in an increase in frequency of abnormal mitotic spindles, misaligned chromosomes and improper spindle dynamics [12]. We have found that phosphorylation of S621 does not directly affect the catalytic capability of MCAK. The variants S621A and S621D show no significant difference in microtubule depolymerization activity relative to the wild type in *in vitro* assays (Figure 4A-C). Nevertheless, *in vivo* analysis of MCAK S621D expressing cells demonstrated an increase in polymerized tubulin (Figure 4D and Figure S2A) and a decreased inter-kinetochore distance compared to WT expressing cells (Figure 5A and B). We suggest that the apparent reduced activity of S621D is due to a reduced cellular protein level as a result of rapid degradation. Measurement of the relative expression of MCAK S621D in cells revealed very low levels compared to MCAK WT and S621A (Figure 5D) by showing an obvious absence of MCAK S621D at certain centromeres/kinetochores (Figure 5C), which provides an explanation for the reduced inter-kinetochore distance observed in these cells (Figure 5A and B). Taken together, the data strongly suggest that the greater amounts of polymerized tubulin and decreased inter-centromere distance observed in cells expressing MCAK S621D is not a consequence of impaired microtubule depolymerization activity but rather of reduced cellular protein levels.

Cells rescued with the non-phosphorylatable mutant MCAK S621A displayed a significant decrease in polymerized tubulin content (Figure 4D and Figure S2A) and in the spindle length relative to WT expressing cells (Figure 4F and Figure S2C). Again this mutant does not show any impairment in depolymerization activity relative to wild type MCAK. Cells expressing this MCAK S621A have much higher levels of the protein relative to MCAK WT expressing cells (Figure 3B and C, Figure 5C and D). We suggest that the observed differences in polymerized tubulin content and in spindle length are due to stabilization of MCAK by this mutation as a result of the inability of the nonphosphorylatable protein to be targeted for degradation. This is consistent with the finding that prevention of degradation, by addition of the proteasome inhibitor MG132, restored a comparable spindle length in cells expressing WT, S621A or S621D (Figure 4G and H).

Taken together, we show that S621 in the C-terminal domain is the major site for phosphorylation of MCAK by Plk1. Phosphorylation at this location does not directly affect its microtubule depolymerization activity but rather likely promotes recognition of MCAK by the Cdc20 subunit of APC/C leading to its proteasome dependent degradation at the transition from metaphase to anaphase (Figure 7A). However, loss of Plk1 phosphorylation on S621 indirectly enhances its depolymerization activity *in vivo* by preventing MCAK's targeting for degradation, resulting in an accumulation of MCAK protein (Figure 7B). By contrast, phosphomimetic MCAK S621D is constitutively targeted for degradation leading to reduced levels of protein and a consequent increase in amount of polymerized tubulin and decreased inter-centromere distance in cells expressing this form of MCAK (Figure 7C). Interfering with this phosphorylation results in defects in spindle formation as well as in chromosome alignment, likely due to the lack of regulation of MCAK protein levels during mitosis.

## MATERIALS AND METHODS

### Cell culture, Western blot analysis, immunoprecipitation and transfection

HeLa and HCT116 cells were cultured as instructed (DSMZ, Braunschweig). HeLa 776-6 cells and HeLa P25 cells were established as reported [33,35]. Cell synchronization, Western blot analysis and immunoprecipitation were performed as described [15,35,58]. Antibodies are listed in Supplementary Information. siRNA targeting the 3'-untranslated region of MCAK was synthesized by Sigma-Aldrich. Control siRNA was purchased from Dharmacon Research Inc. (Lafayette). siRNA and plasmids were transfected as described [35]. Phenotype analysis is detailed in Supplementary Information.

### Immunofluorescence staining, measurement of inter-centromere, the pole-to-pole distance and quantification of the relative expression of EGFP MCAK mutants

Immunofluorescence staining was performed as described [15,58,59] and the primary antibodies are listed in Supplementary Information. The inter-centromere distance and the pole-to-pole distance of metaphase cells were measured using the LAS AF software (Leica, Heidelberg). The immunofluorescence stained slides were further examined by a confocal laser-scanning microscope (CLSM) (Leica CTR 6500). For quantification of the expression of EGFP constructs in cells, the images have been acquired with the confocal laser-scanning microscope (CLSM) (Leica CTR 6500) using a constant exposure. The relative expression of each construct was evaluated using the module “intensity measurements” accessible in the LAS AF software (Leica). Images were further processed using Adobe Photoshop.

### Construction of DNA plasmids, recombinant protein expression and kinase assay *in vitro*

Construction of DNA plasmids and corresponding primers are detailed in Supplementary Information. Recombinant full-length and domains of MCAK were induced and expressed in *Escherichia coli* BL21 (DE3, *Codon Plus*) at 37°C for 2 h by addition of 1 mM IPTG and purified using glutathione-Sepharose 4B beads (GE Healthcare) [35]. Kinase assay *in vitro* was performed as described [15] and briefed in Supplementary Information.

### Microtubule depolymerase activity *in vitro*, ATP hydrolysis assay and measurement of polymerized tubulin *in vivo*

Human MCAK-his_6_ and its mutants were expressed and purified as described [48]. Depolymerization assay and ATP hydrolysis assay were carried as described [15] and briefed in Supplementary Information. Microtubule depolymerization was also monitored in an ensemble assay by light scattering detailed in Supplementary Information. Measurement of polymerized tubulin *in vivo* is described in Supplementary Information.

### Statistical analysis

Student's *t*-test (two tailed and paired) was used to evaluate the significance of difference between the transiently transfected cells with MCAK constructs. Difference was considered as statistically significant when p < 0.05.

## SUPPLEMENTARY INFORMATION AND FIGURES


