# Supplementary material for: Polo-like kinase 1 regulates the stability of the mitotic centromere-associated kinesin in mitosis
Source: Oncotarget. 2014 Mar 24;5(10):3130–44. doi: 10.18632/oncotarget.1861 (PMC4102797; doi:10.18632/oncotarget.1861)
Supplement: Supplementary file 1 [file oncotarget-05-3130-s001.pdf]

Polo-like kinase 1 regulates the stability of the mitotic centromere-associated kinesin in mitosis

SUPPLEMENTARY INFORMATION

MATERIALS AND METHODS

Compounds, antibodies for Western blot analysis and immunofluorescence staining

The Plk1 inhibitor BI 2536, the proteasome inhibitor MG 132 (10 µM) and cycloheximide (25 µM) were purchased from Selleck Chemicals LLC (Houston) and Sigma-Aldrich (Taufkirchen), respectively.

Following antibodies were used for Western blot analysis: mouse monoclonal anti-Kif2c (Santa Cruz biotechnology, Heidelberg), mouse monoclonal anti-Plk1 (Santa Cruz biotechnology), mouse monoclonal anti-cyclin B1 (Santa Cruz biotechnology), mouse monoclonal anti-GST (Santa Cruz biotechnology), mouse monoclonal anti FLAG M2 (Sigma-Aldrich), mouse monoclonal anti-ß-actin (Sigma-Aldrich), mouse monoclonal anti-Emi1 (Sigma-Aldrich), mouse monoclonal anti-phospho-histone H3 (S10) (Millipore, Darmstadt), mouse monoclonal anti-Cdc20 (Santa Cruz), rabbit polyclonal anti-Cdc25C (Santa Cruz biotechnology), mouse monoclonal anti-Cdh1 (Abcam, Darmstadt), rabbit polyclonal anti-Ubiquitin (Cell signaling, Darmstadt), and mouse anti-phospho-Plk1 (T210) (Becton Dickinson, Heidelberg).

For immunofluorescence staining we used following antibodies: rat polyclonal antibody against α-tubulin (Biozol, Eching), rabbit antibody against pericentrin (Abcam), ACA, human anti-centromere antibody (Abcam), rabbit polyclonal antibodies against INCENP (Abcam), mouse monoclonal antibody against Hec1 (Abcam), mouse monoclonal antibodies against Plk1 (Santa Cruz biotechnology), rabbit polyclonal antibodies against EB1 (Millipore). FITC-, Cy3- and Cy5- conjugated secondary antibodies were obtained from Jackson

Immunoresearch (Newmarket). DNA was stained using DAPI (4’,6-diamidino-2-phenylindole-dihydrochloride) (Roche).

Immunoprecipitation and phenotype analysis

Immunoprecipitation was carried out as following: about 800 µg of lysates of Flag-tagged MCAK transfected HeLa cells were filled-up to 300 µl with lysis buffer. FLAG antibody (Santa Cruz biotechnology) and 25 µl Protein G SepharoseTM 4 Fast Flow beads (GE Healthcare, Uppsala) were added and incubated overnight on a rotator at 4°C. The beads were washed three times before SDS-PAGE.

For phenotype analysis, HeLa cells depleted of endogenous MCAK were co-transfected with Flag-tagged MCAK and pBabe-puro constructs (a kind gift from Dr. Liu, Purdue University) at a ratio of 10:1. After selection with 2 µg/ml puromycin, floating cells were washed away and attached cells were cultured for further phenotype analyses including cell cycle analysis, inter-centromere distance measurement, indirect immunofluorescence for assessment of chromosome positioning and abnormal spindle formation. All experiments were independently performed at least three times.

Construction of DNA plasmids

Full-length human MCAK cDNA was obtained from RZPD (IRATp970F0111D, Berlin) and was cloned into BamH1/EcoR1 sites of pGEX 5x-3 (GE healthcare, Munich), into EcoR1/BamH1 sites of p3xFLAG-CMV7.1 (Invitrogen) and into BamH1/EcoR1 sites of pEGFP-C2 (Invitrogen). Various domains of MCAK were also sub-cloned into the pGEX 5x-3 (GE healthcare). Following primers were used for constructing DNA plasmids: N-terminus (aa 1-187): up primer: 5’-atggccatggactcgtcgcttcagg-3’, down primer: 5’-aactgagttcacagggtttgcagaagagct-3’; neck (aa 188-255): up primer: 5’-ctcaggattccggttcggaggaaatcatgtcttgtg-3’, down primer: 5’-aactgagttcacagggtttgcagaagagct-3’;

core (aa 256-590): up primer: 5’-aggaaacgcccactgaataagcaag-3’, down primer: 5’-actgtgggggctcagctccttgaccctgtc-3’; C-terminus (aa 591-725): up primer: 5’-gggcccagtggagagcagttgatt-3’, down primer: 5’- tcactggggccgtttcttgctgcttat-3’. Point mutations were generated with the Quick Change-site-directed mutagenesis Kit (Stratagene, Amsterdam) using the following primers: S621A up: 5´-cgctgattccaggcaatttagccaaggaagagga-3, S621A down: 5´-tcctcttccttggctaaattgcctggaatcagcg-3; S621D up: 5´-gcgctgattccaggcaatttagacaaggaagaggaggaac-3, S621D down: 5´-gttcctcctcttccttgtctaaattgcctggaatcagcgc-3´.

Kinase assay *in vitro*

GST-tagged MCAK and subdomain proteins were incubated with Plk1 kinase (Biomol, Hamburg) in the presence of 1 µCi [γ^{32}P] ATP and 100 µM non-radioactive ATP at 37°C for 30 minutes. The reaction was stopped by adding sample buffer and boiling for 5 min. Equal volumes of each reaction were loaded onto 10% SDS PAGE and separated. The gel was stained with Coomassie and scanned as input control. The incorporation of ^{32}P was quantified using Image J software.

Microtubule depolymerase activity *in vitro* and ATP hydrolysis assay

Depolymerization assay *in vitro* was carried out using GMPCPP stabilized microtubules immobilized in microscope chambers. Assays were carried out in BRB20 buffer supplemented with 75 mM KCl, 1 mM ATP, 0.1 mg/ml BSA, 1% β-mercaptoethanol, 40 mM glucose, 40 µg/ml glucose oxidase, 16 µg/ml, depolymerization was initiated by addition of 40 nM of either MCAK WT, S621A or S621D. The rates of depolymerization of individual microtubules in the field of view were calculated.

Microtubule depolymerization was also monitored in an ensemble assay by light scattering. Depolymerization assays were initiated by addition of 50 nM MCAK WT or its variants to 1 μM polymerized tubulin (GMPCPP stabilized microtubules) in BRB80, 75 mM KCl, 1 mM MgATP, 1 mM DTT, 200 μg/ml BSA. The light scattering signal was measured using a F-2500 fluorimeter (Hitachi), λ_{ex} and λ_{em} = 350 nm readings were taken every 5 seconds. The signal was normalized to the signal for microtubules prior to the addition of MCAK.

The rate of ATP hydrolysis was determined by monitoring the production of inorganic phosphate by the reaction of 3 μM MCAK or its variants with 2 mM MgATP in BRB80, 75 mM KCl. Samples were quenched by addition of an equal volume of 2 M HCl and neutralized by addition of 1 M Tris and 3 M KOH. Liberated phosphate was quantified using the BIOMOL green phosphate detection reagent (Enzo Scientific) and measuring absorbance at 650 nm. The concentration of phosphate in each sample was determined by comparison with a phosphate standard curve. The ATP turnover rate was calculated by dividing the rate of increase in phosphate concentration by the concentration of MCAK motor domains.

Measurement of polymerized tubulin *in vivo*

HeLa and HCT116 cells were depleted of endogenous MCAK using siRNA targeting the 3'-untranslated region of MCAK on day 1 and Flag-tagged MCAK plasmids constructs were added back on day 2. Cells were synchronized with nocodazole and shacked off to collect the mitotic fraction. Mitotic cells were released for 1.5 h in fresh medium and collected for analysis of cellular microtubule polymer content after extraction, fixation and staining for tubulin using fluorescence flow cytometry (Becton Dickinson, Heidelberg). Briefly, cellular soluble tubulin was pre-extracted in a saponin-containing microtubule stabilizing buffer (2 mM EGTA, 5 mM $MgCl_2$, 0.1 M PIPES pH 7.4, 0.2% saponin and 25 nM paclitaxel).

Resuspended cells in microtubule stabilizing buffer were then fixed with an equal volume of a 4% paraformaldehyde solution at 37°C for 15 min. Cells were then washed and stained for α-tubulin with specific mouse monoclonal antibody (Sigma-Aldrich) and fluorescein-conjugated rabbit anti-mouse antibody (Dako, Hamburg). More than 95% of all cells were included in the acquisition gate and 100,000 cells were examined. Fluorescence intensity was quantified using the Cell Quest software (Becton Dickinson). Cells transfected with MCAK WT were assigned as microtubule polymer content 100%. The experiments were independently performed three times and each time was in triplicate.

Supplementary Figures

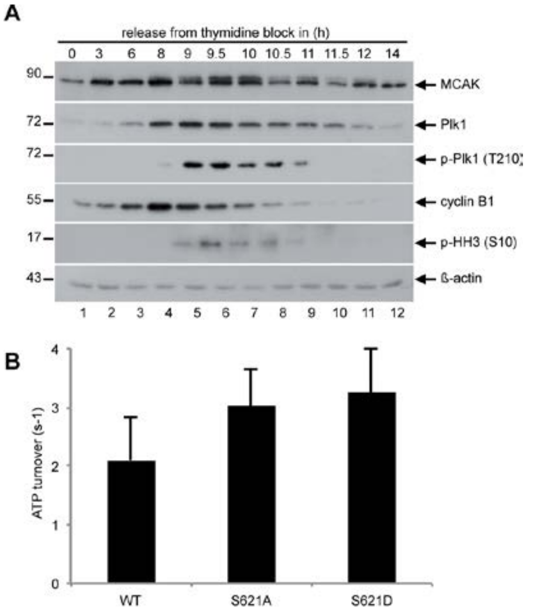

Figure S1: The expression of MCAK and Plk1 is cell cycle regulated during mitosis. (A) HeLa cells were synchronized to the G1/S boundary by a double thymidine block and released into fresh medium. Cell lysates were harvested at the indicated time points and analyzed with the indicated antibodies. (B) ATP hydrolyzation assay. The rate of ATP turnover of MCAK WT (n=4), S621A (n=3) and S621D (n=3) was determined by measuring the production of inorganic phosphate. The results are represented as mean ± SD and statistically analyzed.

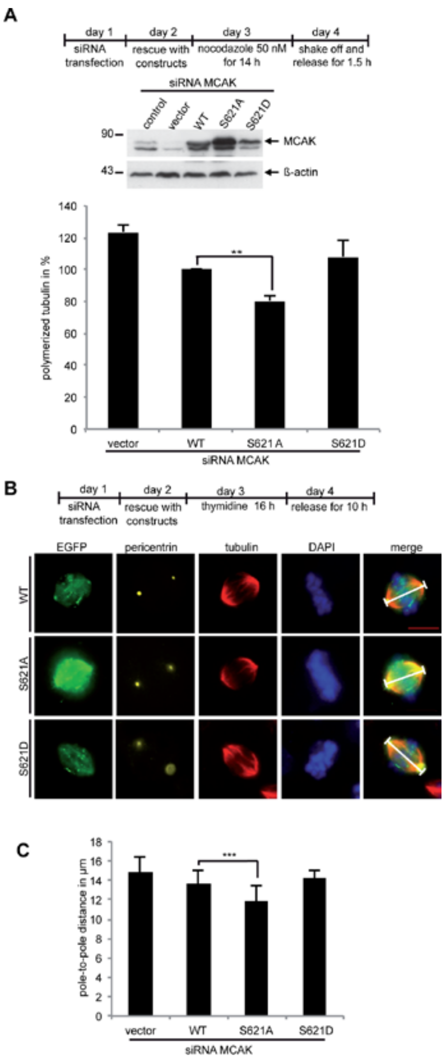

Figure S2: Less polymerized tubulin and shorter inter-centrosome distance in HCT116 cells rescued with MCAK S621A. (A) Measurement of polymerized tubulin content *in vivo*. Upper panel: working schedule. HCT116 cells were transfected with Flag-tagged MCAK WT, its mutants or the empty vector in an endogenous MCAK depleted background. Transfected cells were then synchronized to prometaphase and released for 1.5 h. Middle panel: Western blot analyses were carried out as transfection controls. Lower Panel: Cellular polymerized tubulin contents were analyzed by flow cytometry after cells were extracted, fixed and stained for tubulin. The amount of polymerized tubulin in Flag MCAK WT-transfected HCT116 cells was assigned as 100%. The results are presented as mean ± SD (n=3). **P < 0.01. (B) Measurement of the inter-centrosome distance in HCT116 cells. Upper panel: working schedule. Lower panel: examples of HCT116 metaphase cells rescued with EGFP MCAK and

its mutant EGFP MCAK S621A and EGFP MCAK S621D. Scale bar: 10 µm. (C) Quantification of the inter-centrosome length using the Axiovision software (n=22 metaphase cells for each condition). The results are presented as mean ± SD and statistically analyzed. ***p < 0.001.

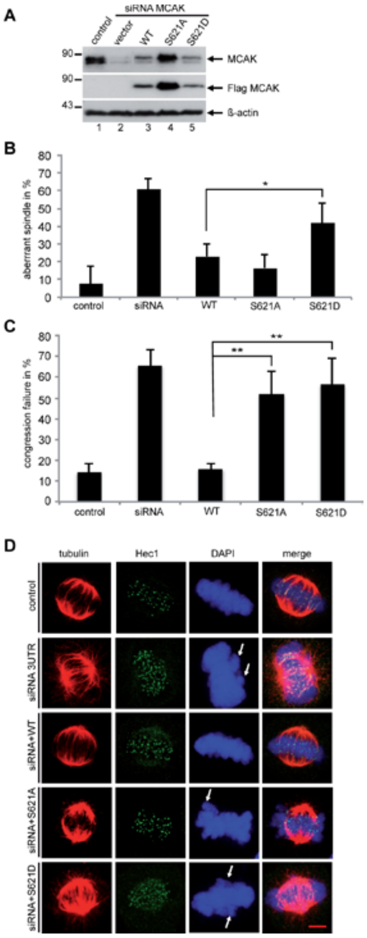

Figure S3: Interfering with Plk1 phosphorylation of S621 in MCAK results in defects in spindle formation and chromosome alignment in HeLa cells. Rescue experiment: HeLa cells were transfected with siRNA targeting endogenous MCAK followed by the rescue with Flag MCAK wild type and its mutants Flag MCAK S621A and Flag MCAK S621D. Cells were synchronized to G1/S by a double thymidine block and released into fresh medium for 12 h. (A) Western blot analysis as control for siRNA and plasmid transfection. (B)Transfected

HeLa cells were stained for tubulin and DNA. The percentage of cells showing aberrant spindles (n=300-500 metaphase cells in each condition). The results are represented as mean ± SD and statistically analyzed. *$p < 0.05$. (C) The frequency of chromosome mispositioning defined microscopically using DAPI stain (n=300-500 metaphase cells in each condition) and is displayed as mean ± SD and statistically analyzed. **$p < 0.005$. (D) Examples of aberrant spindles and chromosome mispositioning in HeLa cells transfected with siRNA against endogenous MCAK and rescued with Flag MCAK and its mutants. Scale bar: 7.5 µm.
